# Recent Developments of PFAS-Detecting Sensors and Future Direction: A Review

**DOI:** 10.3390/mi11070667

**Published:** 2020-07-08

**Authors:** Kelsey L. Rodriguez, Jae-Hoon Hwang, Amirsalar R. Esfahani, A H M Anwar Sadmani, Woo Hyoung Lee

**Affiliations:** Department of Civil, Environmental, and Construction Engineering, University of Central Florida, Orlando, FL 32816, USA; klrodriguez@Knights.ucf.edu (K.L.R.); Jaehoon.Hwang@ucf.edu (J.-H.H.); aesfahani@Knights.ucf.edu (A.R.E.); sadmani@ucf.edu (A.H.M.A.S.)

**Keywords:** per- and poly-fluoroalkyl substances (PFASs), PFAS detection sensor, smart cities, smart sensing technology

## Abstract

Per- and poly-fluoroalkyl substances (PFASs) have recently been labeled as toxic constituents that exist in many aqueous environments. However, traditional methods used to determine the level of PFASs are often not appropriate for continuous environmental monitoring and management. Based on the current state of research, PFAS-detecting sensors have surfaced as a promising method of determination. These sensors are an innovative solution with characteristics that allow for in situ, low-cost, and easy-to-use capabilities. This paper presents a comprehensive review of the recent developments in PFAS-detecting sensors, and why the literature on determination methods has shifted in this direction compared to the traditional methods used. PFAS-detecting sensors discussed herein are primarily categorized in terms of the detection mechanism used. The topics covered also include the current limitations, as well as insight on the future direction of PFAS analyses. This paper is expected to be useful for the smart sensing technology development of PFAS detection methods and the associated environmental management best practices in smart cities of the future.

## 1. Introduction

Perfluoroalkyl and polyfluoroalkyl substances, commonly referred to as PFASs, are largely man-made chemicals that are hydrophobic and lipophobic [1,2]. Applied widely as industrial chemicals and in consumer products, PFASs’ unique properties are useful for their durability and resistance to heat, oil, and water. Unfortunately, recent awareness has brought attention to the toxicity of these substances. PFASs are associated with health risks, such as cancer, infertility, low birth weight, and delayed puberty [2,3]. In particular, dyslipidemia, a disorder affecting lipid production, has one of the strongest metabolic correspondences to PFAS exposure [4]. Their toxicity to humans and other organisms has incurred interest regarding regulating concentrations, as well as developing determination and treatment methods.

PFASs are highly fluorinated aliphatic substances. Their C–F bond is one of the strongest found in nature and becomes stronger with increasing hydrogen replacement by fluorine at each carbon. Due to their stable chemical structure, high electronegativity, and the small size of the fluorine atom, PFASs represent a class of environmentally persistent substances with long biological half-lives and a high accumulation potential [5]. The unique properties of fluorinated compounds present challenges for current analytical techniques, which is motivating the recent surge in research supporting PFAS determination and treatment technology.

A diverse mixture of PFASs in varying concentrations can be found in many areas of daily and industrial use. These substances are well-known constituents of products, such as fire-fighting foams, metal plating, lubricants, paints, polishes, and food packaging [6]. Fire-fighting foams are a particular source of concern since large quantities are used in liquid form during a relatively short period, increasing the risk for groundwater contamination [7]. PFAS concentrations have also been found in products such as ski waxes (up to about 2000 μg/kg perfluorooctanoic acid (PFOA)), leather samples (up to about 200 μg/kg pentafluorobenzoic acid (PFBA) and 120 μg/kg perfluorobutane sulfonate (PFBS)), outdoor textiles (up to 19 μg/m^2^ PFOA), and some baking papers (up to 15 μg/m^2^ PFOA) [8]. Due to their toxicity, curiosity regarding the environmental release of PFASs and their life cycle has arisen. In the life cycle of products containing concentrations of PFASs, landfills are typically considered the final stage [9]. This introduces contamination potential in solid wastes, leachates, landfill gas, biosolids, and groundwater, which is more difficult to phase out than direct sources of exposure [4]. A single study has reported that the global distribution of PFAS concentration ranges in landfill leachate is between 0.1 and 250,000 ng/L [9]. The potential for low concentrations and wide ranges add yet another source of challenges in current analytical techniques.

There are many families of PFASs with numerous corresponding homologs and isomers. “Long-chain” perfluoroalkyl sulfonic acids (C_n_F_2n+1_SO_3_H, n ≥ 6, PFSAs) and perfluoroalkyl carboxylic acids (C_n_F_2n+1_COOH, n ≥ 7, PFCAs) and their corresponding anions are typically more bioaccumulative than their short-chain analogs [1]. Their resistance to degradation and higher accumulation potential make perfluorooctane sulfonate (PFOS) and PFOA two of the species among the long-chain perfluoroalkyl acids that are most often investigated. In 2002 and 2015, the U.S. Food and Drug Administration (FDA) banned PFOS and PFOA, respectively, from food packaging. Similarly, the US Environmental Protection Agency (USEPA) has initiated actions against PFASs. For example, in 2006, the USEPA worked with eight leading chemical companies to reduce PFOA by 95% through the PFOA Stewardship Program [9]. In 2016, the USEPA released health advisory levels of 70 parts per trillion (ppt) for PFOS and PFOA, individually and combined [10]. Following the emergent efforts taken against PFASs, the USEPA released a PFAS Action Plan in 2019 [11]. The action plan defines the steps the agency is taking to address PFAS and to protect public health, and a November 2020 target was set to identify PFOS and PFOA as hazardous substances under the Superfund law [12].

In this study, as PFASs contamination has been a major disruption in environmental management, the current methods for the analysis of these emerging contaminants in environmental matrices were reviewed. First, the traditional technologies most used are discussed, which include liquid chromatography–mass spectrometry (LC-MS) [13,14,15] and gas chromatography–mass spectrometry (GC-MS) [16,17,18]. However, their limitations in practical applications have motivated a different direction in the literature. To adhere to the evolving regulations, this disruption in environmental management requires higher levels of versatility and reliability regarding the determination of PFASs in environmental samples than produced by methods that are currently available. With general success in the field of sensor applications in environmental matrices containing various constituents, PFAS-determining sensors have become the focus in the literature. Sensor technology, compared to traditional methods, most significantly allows for in situ detection, ease of use, and low-cost operations. These factors are essential for developing an overarching and integrated “smart” environmental management system. Thus, critical evaluations of sensor development, application, and data interpretation for PFAS determination were conducted in addition to the review of traditional methods. We aimed to classify sensor technologies in terms of their detection properties and the quantity of PFAS to be measured.

## 2. Overview of Existing Technology

### 2.1. Current Methods for the Analysis of Per- and Poly-Fluoroalkyl Substances (PFASs) in Environmental Matrices

Table 1 shows the currently available analytical tools for PFAS measurements in various environmental matrices. PFAS analysis is predominantly based on high-performance liquid chromatography (HPLC) coupled with tandem mass spectrometry (MS/MS), although GC-MS and LC-MS have also been used for the analysis of selected PFASs in some cases (Table 1). Some researchers have used orbitrap or time-of-flight (ToF) MS for quantitative and qualitative analyses [19]. Depending on the molecular characteristics of the target PFASs and the type of information required from the analysis, electrospray ionization (ESI), atmospheric pressure chemical ionization (APCI), or atmospheric pressure photoionization (APPI) have been applied for PFAS measurements [19,20].

PFASs from various aqueous matrices are typically extracted using solid-phase extraction (SPE) [21,22,51,52], but other methods, including liquid–liquid extraction (LLE) [23], ion-pair extraction (IPE) [24], solid-phase microextraction (SPME) [25], and dispersive liquid–liquid microextraction (DLLME) [26], have also been used in many studies. The most common protocol for off-line SPE (typically polystyrene divinylbenzene contained cartridges) involves extract elution using methanol followed by concentration to dryness with nitrogen before injecting the sample into the column and analyzing it using LC-MS/MS [53,54]. Water matrices pose significant challenges to the analysis of organic pollutants at very low concentrations (usually sub ng/L levels), causing poor analytical accuracy and reproducibility [55]. SPE offers both sample extraction and cleanup, and hence, it is the preferred method when analyzing PFASs in various water matrices. In this context, it is necessary to determine method detection limits (MDLs) for the specific water matrix in which the PFASs are analyzed. Surma et al. [27] developed a method to determine PFOA and PFOS concentrations in honey samples using dispersive solid-phase extraction (d-SPE) with a recovery of up to 87% that was analyzed using a micro-ultrahigh performance liquid chromatography (UHPLC)-MS/MS. The d-SPE method requires a small amount of sorbents that are dispersed in the aqueous solution, while offering high compound recoveries, low usage of solvent, and utilization of the maximum sorbent surface area [27,54], unlike the SPE cartridges, which may clog when extracting a relatively polluted water matrix. Hence, d-SPE should be explored further for PFAS analysis in a range of water matrices. Recently, Deng et al. [28] explored bamboo charcoal as an SPE medium to extract selected perfluoroalkyl acids (PFAAs) in ultrapure water, drinking water, and surface water samples that were analyzed using LC-MS/MS. The bamboo charcoal-based SPE demonstrated low LODs (0.01–1.15 ng/L) and good repeatability and reproducibility (5.3–8.0%, n = 3).

Micro-SPE (µSPE) is an alternative technique that allows for the use of smaller sorbent particle sizes (<5 µm) when operating under high pressure, requiring a reduced volume of sample extraction and an increased extraction efficiency [56]. Recently, Lockwood et al. [29] used µSPE cartridges packed with mixed-mode C18:aminopropyl silica (APS) phase to analyze 13 long- and short-chain PFASs in surface waters and demonstrated similar result to conventional SPEs while reducing the sample volume and preparation time to 2 mL and 5 min, respectively [29]. The authors reported PFAS concentrations of up to approximately 900 ng/L in the tested surface waters with recoveries ranging from 86% to 111%. Villaverde-de-Sáa et al. [24] compared two polymeric materials, namely, polydimethylsiloxane (PDMS) and polyethersulfone (PES), for sorptive extraction of selected perfluorinated compounds in river water, seawater, and sewage samples. They reported better sensitivity using PES when compared to PDMS, with LODs ranging from 0.2–20 ng/L depending on the water matrix.

Manual SPE is labor- and time-intensive and often requires a large sample volume to reach the desired limits of detection (LOD). Solid-phase microextraction (SPME) is a fused-silica-fiber-based extraction method that saves preparation time, solvent usage, and disposal costs, as well as facilitates a lower LOD when extracting volatile and semi-volatile organic compounds from environmental samples [57]. Saito et al. [30] developed an on-line in-tube SPME method that was coupled with LC-MS to determine PFOS and PFOA concentrations in tap water and river water samples. This method allowed for convenient automation of the extraction process, resulting in a reduced analysis time while yielding better precision and sensitivity when compared to manual extraction. The detection limits for PFOA and PFOS following this protocol were 1.5 and 3.2 ng/L, respectively [30]. Bach et al. [31] validated a headspace solid-phase microextraction (HS-SPME) method to analyze selected volatile PFASs in water and sediment samples using GC-MS. The authors reported a limit of quantification (LOQ) of 20–100 ng/L with recoveries ranging from 76–126% in water samples, depending on the PFAS and water matrices. This simple, rapid, and solvent-free method helped to reduce the sample preparation time, allowing for the simultaneous analysis of a large number of samples in various environmental matrices [31].

Another simple and rapid analytical method that was applied for the analysis of perfluorocarboxylic acids (PFCAs) in aqueous matrices at low concentrations was developed by Alzaga and Bayona [18], who utilized tetrabutylammonium (TBA) as an ion pair in conjunction with SPME followed by in-port derivatization–GC–negative ion chemical ionization MS. The authors reported a reduced analysis time, reduced sample and solvent volume, and improved recoveries when using this method, and hence, suggested its application toward the rapid screening of PFCAs in environmental samples. For the efficient extraction of PFCAs in complex water matrices, Huang et al. [25] recently proposed a novel multiple monolithic fiber solid-phase microextraction (MMF-SPME) approach that follows the same extraction protocol as conventional SPME; however, this process possesses a higher extraction capacity due to MMF and offers a low-cost, simple operation with a fast mass transfer. The authors reported the LOD of the target PFCAs in tap water, river water, wastewater, and milk samples to be 0.40–12.1 ng/L [25].

In water containing a high amount of particulate matter (e.g., wastewater), the PFASs may adsorb to the particles [58]. Hence, filtering the water before the SPE of PFAS from such water matrices may result in low recovery due to compound losses during the filtration step, in addition to possible losses on the walls of the sample containers [23,59]. Such losses can be avoided by using liquid–liquid extraction (LLE) directly from the container. Wang et al. [26] applied a rapid and highly selective method for PFAS extraction in aqueous solutions using dispersive liquid–liquid microextraction (DLLME). This fluorous affinity-based extraction method utilizes less extraction solvent while minimizing the water matrix effects, achieving over 70% recoveries for selected medium- and long-chain PFASs (CF_2_ > 5) [26]. Papadopoulou et al. [32] developed a vortex-assisted liquid–liquid microextraction (VALLME) method for the rapid screening of PFOS in water. In this method, the vortex mixing of microvolumes of the extractant solvent in the sample resulted in fine droplets extracting the target analytes, which were subsequently separated via centrifugation and analyzed using LC-MS. The authors reported relative standard deviations of 7.4% and 6.5% for 10 and 500 ng/L of PFOS spiked in tap water, river water, and groundwater samples, and observed that the water matrices did not affect the extraction [32].

Recently, Goh and Lee [33,60] developed a bundled hollow fiber array (BHF) liquid-phase microextraction (LPME) method to exploit the volume capacity of the HFs’ pores, whereby the BHFs serve as a solid support to immobilize the extractant solvent and facilitate contact with the aqueous sample over a large area. While low relative standard deviations (RSD < 12%) were observed for selected perfluorinated compounds using this method, the relative recoveries were markedly variable (approximately 9 to 70%), which was attributable to the water matrix effects [33]. This automated, rapid method of extraction can be employed as an on-site water quality monitoring system when perfluorinated compounds are of interest.

As discussed above, several extraction and analytical techniques have been developed for a wide range of PFASs. However, as per a survey conducted by the Swedish Chemicals Agency, there are probably more than 3000 PFASs currently on the global market [61]. The occurrence of the large quantity of currently detected and future perfluorinated compounds makes it a very challenging task to monitor them in environmental matrices, requiring the time- and/or resource-consuming analytical methods to keep pace with the “never-ending” chemicals [62]. For instance, the USEPA first developed an SPE-LC-MS/MS method (Method 537, Rev 1) for the determination of 14 PFAAs in drinking water [51]. This was followed by an updated method (Method 537.1) in 2018 to include four more PFAAs [63]. Very recently (November 2019), the USEPA has published a new validated method (Method 533) that compliments Method 537.1 and can now measure 29 PFASs, focusing on the “short-chain” PFASs with four to twelve carbon chain lengths [64]. In response to the growing number of perfluorinated chemicals currently found in biological and environmental media and to be characterized in future, the determination of total organic fluorine (TOF), which is a total index of water quality, can serve as an alternative method to screen environmental matrices for the presence of PFASs [20,59,65]. Several studies have demonstrated TOF analysis via combustion ion chromatography (CIC) [65,66,67] and sodium biphenyl (SBP)-based defluorination methods [20,68]. In CIC, samples are combusted at 900–1000 °C to convert organic fluorine to hydrofluoric acid, which is absorbed in an alkaline solution, followed by the identification of fluoride ions (F^−^) in that solution using IC [65,66]. In the SBP method that was developed to covalently bind fluorine in organic compounds and biological materials [68], the F^−^ released following the reaction with SBP is detected using a fluoride-ion-selective electrode (ISE) [20,69], or via a flow-injection system with either fluorimetric or potentiometric detection [70]. Very recently, a comprehensive analytical workflow for the assessment of organofluorine (OF) has been proposed by Koch et al. [70], where the authors also suggest that the OF methods could be integrated with target analysis, suspect screening, or non-target screening for further identification.

### 2.2. Challenges and Limitations of Existing Analytical Methods of PFASs

The conventional analytical methods for the detection of PFASs are LC-MS [13,14,15] and GC-MS [16,17,18]. These methods utilize the same technique based on ion-pair extraction of the analytes and quantification by MS, and can detect concentrations as low as ppt. However, to summarize some of their most significant limitations, these methods typically require off-site analyses, are very time consuming, and matrix-matched calibration standards are not routinely employed. One of the more prominent setbacks in chromatographic systems includes the problem of peak separation with PFOA and PFOS due to the blank originating from ubiquitous polymeric parts; thus, PFOA could be retained and eluted after the injected PFOA [71]. While LC-MS has the advantage of better separation compared to GC-MS, GC-MS needs to be combined with electron impact (EI) or chemical ionization (CI), which offers the advantage of the applicability of mass spectral libraries. However, GC-MS also offers similar disadvantages to LC-MS, such as time-consuming sample analyses and being too expensive for common applications in the field of environmental monitoring.

Work completed by Lan Liu shed light on the prominent limitations of LC-MS by comparing capillary liquid chromatography–MS (CLC-MS) and ultrahigh-performance LC–tandem MS (UHPLC-MS/MS) [72]. Although these methods have demonstrated reliable results, substantial challenges still exist in increasing the number of PFASs detected and quantified in a single analytical run, working with varied sample matrices, and developing more efficient sample preparation strategies. Recovery for the UHPLC-MS/MS was greater than 100%, suggesting that there was a source of carry-over contamination during multiple sample analyses.

Tandem MS also suffers from considerable sensitivity loss due to low fragmentation yields for some PFASs [73]. A study by Berger et al. compared four types of mass spectrometers (ion-trap MS, time-of-flight high-resolution MS (ToF-HR-MS), quadrupole MS, and triple quadrupole MS) for PFAS analysis. HPLC ToF-HR-MS was suggested as a more sensitive alternative technique, with a detection limit that is an order of magnitude lower compared to tandem MS, although hindered by small linear ranges and the lack of ability for MS/MS, which provides useful quantification information [73]. The ion-trap MS was also limited by small linear ranges, as well as high LODs and a low mass cut-off. However, due to the low distribution of ToF-HR-MS technology in analytical laboratories, quadrupole MS/MS is most frequently used. Enduring limitations of these technologies make the development of easy-to-operate, inexpensive, and sensitive assays essential for PFAS detection.

An additional analytical challenge is caused by the limited amount of PFASs (≈28) currently being analyzed among the abundant quantity of species that exist in the global market (>3000) [74]. Many of these substances are considered unknown and labeled as “PFAS precursors”, meaning that they can degrade to form PFASs. Therefore, the structures of each precursor may be unknown and/or there may be an absence of standards, making detection with the current methods difficult.

## 3. Needs and Current Status of PFASs Sensor Development

Detection of PFASs in the aqueous environment is limited as a result of relatively low concentrations in the order of ppt; these are most commonly detected using HPLC coupled with electrospray-ionization MS, which can only be found in professional laboratories [75]. Though this method is capable of detecting model concentrations found in nature, the drawbacks include the high associated costs (>$100 per sample), off-site analysis, and time-consuming efforts. The development of sensors to detect contaminants in environmental samples is a growing topic in environmental monitoring and management. Many limitations within existing methods of PFAS determination can be addressed through the development of PFAS-detecting sensors. With the increasing urgency in developing versatile and reliable detection methods, a trend in sensor development for PFAS determination was investigated in the literature. In more recent years, research has been focused on developing on-site detection methods, such as ion-selective electrode (ISE) [76,77], electrochemical sensors [78,79,80], fluorescence sensors [79], and smartphone app-based monitoring systems [75], that are both reliable and more economically feasible than conventional methods. Examples of these detection methods, along with their LODs, are outlined in Table 2.

### 3.1. Electrochemical Sensors

**Figure 1:**
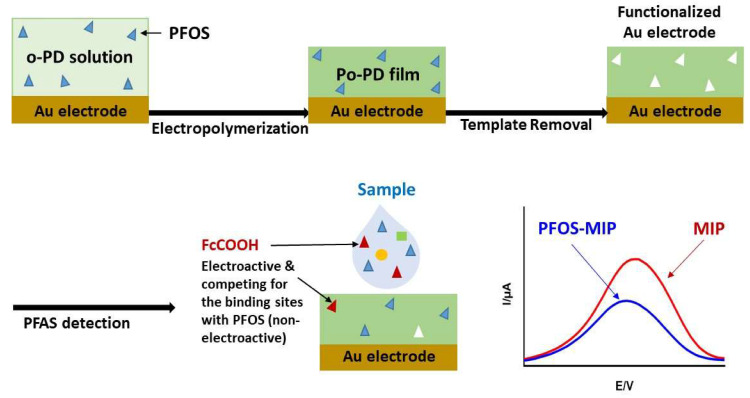
Schematic diagram of molecular imprinting and PFAS detection on a gold electrode. MIP: molecularly imprinted polymer, o-PD: *ortho*-phenylenediamine, Po-PD: poly(*o*-phenylenediamine). Adapted with permission from Karimian et al. [78].

Various classes of electrochemical sensors exist and are categorized based on their electrical magnitude of detection: potentiometric measures changes in the ion-selective membrane potential (mV), conductometric measures changes in the conductance (G, Ω^−1^), impedimetric measures changes in the impedance (Z) over a range of frequencies (Hz), and voltammetric measures the change in current (pA) that occurs as a result of the initial electrochemical reaction caused by an applied voltage (mV). Various types of electrochemical sensors have been used to detect chemical and biological compounds, such as heavy metal ions [86,87,88,89,90,91], nitrite [92], pH [93,94], dissolved oxygen (DO) [95,96], phosphate [96,97,98,99], and free chlorine (or monochloramine) [94,100,101,102,103]; the technology used ranges from microelectrodes to screen-printed electrodes and can be applied to natural and engineered water systems and environmental samples (e.g., biofilms, metals, and plants). Voltammetric and potentiometric sensors are the most common types of electrochemical sensors used for PFAS detection. However, to utilize these electrodes, the surfaces must first be functionalized such that they can directly interact with the target analyte through ion exchange or complexation. This can be achieved through the use of molecularly imprinted polymers (MIPs), which provide a polymeric matrix on the surface of the electrode with voids, or recognition sites, that are complementary to the shape, size, and functional groups of the target analyte (Figure 1) [78].

**Figure 2:**
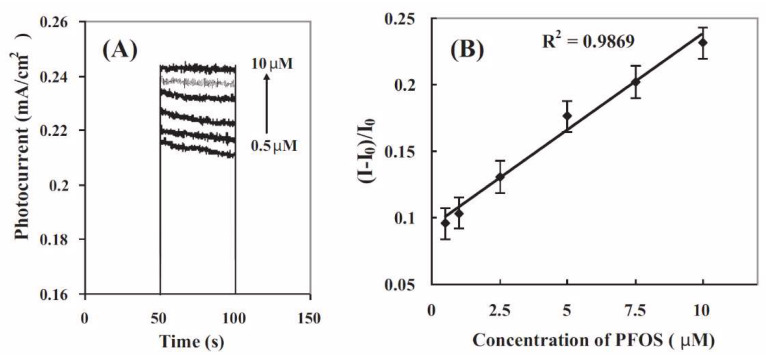
Photocurrent responses of (**A**) an MIP@TiO_2_ nanotube arrays (NTA) electrode in 0.1 M PBS (pH 7) solution containing 0.5, 1, 2.5, 5, 7.5, and 10 µM PFOS (from bottom to top). (**B**) Linear calibration curve. All photocurrents were recorded after the electrodes were immersed in the PFOS solution for 15 min. Reproduced with permission from Tran et al. [81].

MIPs may provide conductive properties, as well as a stable technique suited for micromachining and integration that has a low cost of production [104]. In recent years, MIPs have been applied for the detection of PFOSs in water [78,81], though the lack of electrochemical activity of this specific analyte (i.e., PFOS) has proven a challenge. To remedy this, Karimian et al. utilized ferrocenecarboxylic acid (FcCOOH), which acts as an electroactive reporter molecule competing with PFOS (non-electroactive) for the MIP sites (Figure 1) [78]. In the presence of PFOS in the sample, the voltammetric signal decreases and a relationship between the PFOS concentration and the signal is developed. It was found that the voltammetric signal of this reporter molecule, FcCOOH in this case, was inversely proportional to the concentration of PFOS in solution. Tran et al. developed a photoelectrochemical PFOS sensor that consists of molecularly imprinted polyacrylamide on vertically aligned TiO_2_ nanotubes that detect PFOSs by measuring the increases in photocurrent that result due to interactions between PFOS and the MIP coating [81]. Unlike the results obtained from Karimian et al., in which the observed signal is inversely proportional to the concentration of PFOS in their water sample [78], Figure 2 shows the results achieved by Tran et al., in which an increase in PFOS concentration corresponds to an increase in the photocurrent observed [81] from different sensing mechanisms. Though efficient, this as-prepared sensor has a LOD of 86 ng/mL^−1^, which is higher than concentrations typically found in the natural environment.

A study by Cheng et al. focused on two significant limitations of electrochemical affinity sensors that currently make them impractical as first response devices in the environmental management of PFASs [85]. These limitations include issues with the transduction step and a low sensitivity when measuring inherently trace concentrations. The transduction step requires the target analyte to reach the recognition element, which results in long detection times (hours). In addition, to improve an often-compromised transducer signal, bulky and expensive instrumentation is typically required. Cheng et al. began addressing the need for an ultrasensitive detection technique that is suited for first-response devices by embedding metal-organic framework (MOF) capture probes on a microfluidic platform between interdigitated microelectrodes (IDμE) with the intention of increasing the sensitivity of the impedance changes observed [85]. The synergistic effect provided by the mesoporous probes and the microelectrodes ensured penetration of the electric field across the entire platform and allowed for interaction with the PFOS at a molecular level, capturing minute changes in interfacial charge transport at any position within the channel. Furthermore, the authors found that this approach led to a significant increase in the signal-to-noise ratio.

**Figure 3:**
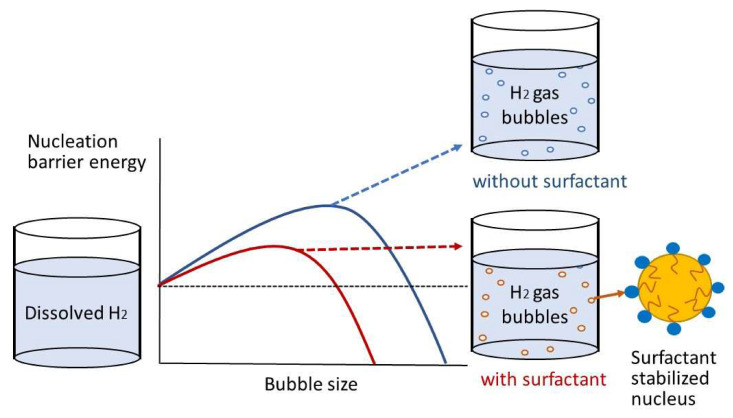
Schematic diagram of the bubble-nucleation-based electrochemical method for PFAS detection. Adapted with permission from Ranaweera et al. [80].

However, to avoid complications associated with low electroactivity, a bubble-nucleation-based electrochemical method that detects concentrations of PFASs based on their high surface activity has also been proposed [80]. The method, proposed by Ranaweera et al., consists of applying a sub-50-nm Pt nanoelectrode to an acidic solution, which causes hydrogen evolution reactions (HERs), resulting in a measurable current upon negatively scanning the nanoelectrode potential until it reaches a peak value (*i*_peak_) [80]. As shown in Figure 3, the sudden drop in the HER current past the peak corresponds to the formation of an H_2_ gas bubble at the electrode, thereby blocking the electrode surface. The presence of PFASs in solution reduces the surface tension of the gas–liquid interface, thus reducing the nucleation barrier due to H_2_ bubble nuclei stabilization. Therefore, in this method, an electrochemical transducer reports changes in the surface tension of the gas–liquid interface, which is proportional to the PFASs’ concentration due to their effect on the stabilization of gas nuclei.

Potentiometric detection of these fluoro-surfactants has also been recently demonstrated. For instance, Fang et al. utilized the MIP technique and pencil lead as an electrode material to detect concentrations of perfluorooctanoic acid (PFOA), PFOS, and 1H,1H,2H,2H-perfluorooctanesulfonic acid (6:2FTS) in the range of 10 µM–10 mM. It was discovered during experimentation that the selectivity of the PFOA-MIP for PFOA was higher than others due to its small recognition site. Another successful demonstration of potentiometric detection was performed by Chen et al., who utilized ion-selective electrodes (ISEs) with fluorous anion liquid exchange membranes (LIX) to detect perfluorooctanoate (PFO^−^) and PFOS with a low LOD of 0.07 µg·L^−1^ [77]. However, when these ISEs were applied to a native New Jersey lake, it was discovered that the presence of other perfluorinated anions that differed only in their number of carbon atoms hindered the selectivity of this method. This finding suggests that, while these electrochemical sensing technologies offer low LODs for their respective target analytes, their sensing is often limited to only one analyte or may have interference with other similar molecules, and can therefore primarily be used as pre-screening tools (i.e., first-response devices) rather than for multianalyte detection in water bodies.

### 3.2. Optical and Fluorescence Sensors

Table 3 shows a summary of the various optical and fluorescence sensor for PFAS detection. Fluorescence quantification of PFOS has served as another successful means of PFAS detection. Proposed by Feng et al., an MIP fluorescence sensor in the form of MIP-capped silicon dioxide (SiO_2_) nanoparticles anchored with a fluorescent dye was able to detect PFOS concentrations in water as low as 5.57 µg·L^−1^ [79]. Upon the binding of PFOS to the recognition sites of this sensor, fluorescence quenching occurred due to the electron transfer between the dye (fluorescein 6-isothiocyanate (FITC)) and PFOS, thereby reducing the fluorescence emission of the dye, which was easily measured using a fluorescence spectrophotometer. A drawback to this method is that PFOS binding on the surface only occurs under extremely acidic conditions (pH ≈ 3.5), thus increasing operational costs and potential environmental hazards. Additionally, the basic amine groups on the surface of the sensor tend toward protonation in acidic environments, thus reducing the selectivity in solutions that contain various perfluoroalkyl homologs [79].

Like that of fluorescence sensors, optical sensing techniques often utilize organic dyes, though they are less reliant on analytical devices, such as fluorescence spectrophotometers, as detection can often be performed with the naked eye. Optical sensors can rapidly and efficiently detect anions; therefore, they have recently been considered as one of the most practical detecting methods for PFASs. Put simply, PFOA and PFOS are anionic surfactants that can interact with a cationic dye (e.g., methylene blue or ethyl violet) to form an ion pair [75]. For anion sensors, different types of organic dyes were developed based on visible color changes. Plastic optical fibers (POFs) are well known for their successful use as optical fiber sensors. POFs provide flexibility, easy manipulation, and a larger diameter [112]. For instance, a study by Cennamo et al. presented a D-Shaped POF platform based on a specific MIP, which demonstrated a limit of detection (LOD) of 0.5 ppb for PFASs [105]. Various optical sensors (e.g., polymer-gold nanoparticle (AuNP) and self-assembled monolayer (SAM)-AuNP sensors) also reported PFOA detection that was monitored via the colorimetric reaction, where LODs of 100 ppm and 10 ppb were achieved, respectively [106,107]. The previous references also take advantage of nanoscale materials. Nanoparticle-based sensors used for PFAS identification have emerged due to their unique behaviors (aggregation, disaggregation, adsorption, and desorption), small size (1 to 100 nm), and outstanding sensitivity in the form of portable, cheap, and reliable devices [74].

Another type of detection method for PFASs involves a combined carbon nitride (C_3_N_4_) nanosheet with an MIP, which demonstrated a higher PFOA sensing sensitivity and a low LOD (10 ppt) using the electrochemiluminescence (ECL) method [110]. Quantum dots (QDs) have also been developed for the detection of PFOA with a LOD of 120 ppb when fluorescent detection was employed [111]; however, the illumination detection using a photomultiplier tube limits the on-site application of this method. Optical sensing methods still lack certain characteristics and have limitations, thus necessitating the need for further research and development aimed at increasing their selectivity and sensitivity for PFAS detection such that they compare to laboratory-based results. For example, co-existing ions may cause interference in optical characteristics detected by optical-based sensors [113]. Adding a pretreatment step to avoid this interference hinders some benefits that many optical sensing methods provide, such as their simplicity and low cost.

### 3.3. Biosensors

Another interesting sensor concept, demonstrated by Zhang et al., is an electrochemical biosensor that detects PFOS based on PFOS inhibition of the biocatalysis process of an enzymatic biofuel cell (BFC) [83]. The one-compartment BFC developed by Zhang et al. comprises multi-walled carbon nanohorn (MWNH) modified glassy carbon electrodes (GCE), which are used for both the bioanode and biocathode substrate, and glutamic dehydrogenase (GLDH) and bilirubin oxidase (BOD), which are used as the bioanode and biocathode biocatalysts, respectively. Within this design, the presence of PFOS affects the bioactivity of the biocatalysts at both the bioanode and biocathode, resulting in a decrease in the open-circuit voltage of the BFC. The biosensor showed a good correlation of R^2^ = 0.976 between PFOS and the decrease in voltage. In addition, this study incorporated the potential effects on PFOS detection of four perfluorinated chemicals with similar structures to PFOS and two types of chemicals (SMNBS and SDS) that may co-exist in micro-polluted environments. The four perfluorinated substances include perfluorooctanoic acid (PFOA), nonafluorobutanesulfonic acid potassium (PFBSK), perfluorooctanesulfonamide (PFOSA), and heptadecafluorononanoic acid (PFNA). It was shown that the electrochemical biosensor exhibited good selectivity (relative standard deviation from 3.6 to 7.7%) for PFOS against these chemicals, even in real water samples obtained from a local river and reservoir in Dalian, China.

Cennamo et al. took a different approach to biosensors by developing a configuration that includes a platform functionalized with a bio-receptor [84]. The proposed biosensor is characterized by a surface plasmon resonance (SPR) platform based on plastic optical fibers (POFs), together with a bio-receptor for the detection of PFOA and PFOS. First, the platform was modified with an α-lipoic acid compound through the formation of a self-assembling monolayer (SAM). Then, it was derivatized with an ad-hoc-produced mono-specific antibody against PFOA. In this bioassay method, PFOA compounds are covalently attached to an immunological protein carrier (BSA) with a high affinity and selectivity. By increasing the PFOA concentration, and thus the produced antibodies, a decrease in the refractive index value of the receptor layer is observed. The assay’s LOD was less than 0.21 ppb (in seawater). The study expanded their investigation by studying the interactions between the produced antibodies and PFOS; it was found that PFOS exhibited a similar response to PFOA such that the antibodies could also be used to monitor PFOS molecules [84]. Other studies have demonstrated similar results with the bioassay method in which PFOS binds to a specific enzyme (e.g., peroxisomal proliferator-activated receptor-alpha (PPARα)), where a detection range of 2.5–7.5 ppt at a wavelength of 605 nm is achieved [108,109].

## 4. Future Direction of PFAS Detection Analyzers and Sensors

The future direction of PFASs analysis in environmental matrices has shifted from laboratory-based determination toward the use of cost-effective in situ sensors. Based on the relevancy of the literature reviewed with this aim in mind, the sensor techniques of PFAS detection presented in this review are primarily intended to contribute to developing colorimetric enabled PFAS detection strategies, such as through steric hindrance by polymers, bioassays, and self-assembled monolayers. The detection of PFASs can be enhanced using colorimetric techniques, where more sensitive and selective PFAS separation or pre-concentration steps can take advantage of the widening properties of PFAS analysis at low concentrations. It is expected that sensor detection techniques for PFAS analysis that provide both the testing kit and portable device will receive more attention regarding rapid on-site assessments. Particularly, the capacity of polymer or bioassay sensors will continue to be improved for enhanced sensitivity over a wide range of PFASs of environmental concern. The review on the development of PFAS analysis methods, as well as the perspectives discussed herein, are intended to provide insight into the future direction of this field.

### 4.1. Sensor Technology

Methods of in situ analyses can allow for quick analyses and simple operation, mitigate sample transformations that occur during transportation from on-site to the laboratory, and moderate the cost of use. Some specific areas still need improvement and further development, such as (1) certified reference colorimetric methods for PFAS detection are not yet available, impeding long-term method performance and accuracy control; (2) for certain applications, such as the determination of PFAS concentrations in a remote open field (e.g., ocean, river, or non-point pollutant source), better detection limits are required for reliable quantification; (3) more reliable quantification at ultra-trace levels is required for PFAS detection, as well as improved sensor sensitivity and selectivity; and (4) to develop a gas detection sensor for PFCAs because they can volatilize from water surfaces into the gas phase via aerosols.

Additionally, one of the main challenges that limit the continuous monitoring of water characteristics using sensor technology is fouling [114]. Water quality monitoring is intended to be a long-term activity that is used to detect changes over time, making sensors extremely susceptible to fouling. This supports the need for constant calibration and cleaning as the fouling decreases the lifetime of the sensor. Biofouling is particularly concerning for sensors applied in aqueous environments because it often decreases the sensitivity and interferes with readings in a short amount of time. Addressing limitations brought about by fouling can aid in sensor performance and reliability, as well as detecting sudden changes in water quality, daily fluctuations, and long-term trends. Although many PFAS sensors are not currently developed enough to experience this limitation, it is one of the criteria that must be considered when choosing which sensor methodology will be utilized and when discussing the future direction of this field. For instance, sensor technology based on optical properties may be more susceptible to fouling than sensors based on electrochemistry, thus affecting decision-making [115]. One of the solutions for fouling problems related to sensor operations and lifetimes is to simply replace the sensor with a new one when a significant sensor drift is observed. To reduce labor costs and related maintenance issues, the sensors can be designed in the form of a replaceable sensor cartridge or revolver type of sensor platform.

### 4.2. Smart Sensing Technology

**Figure 4:**
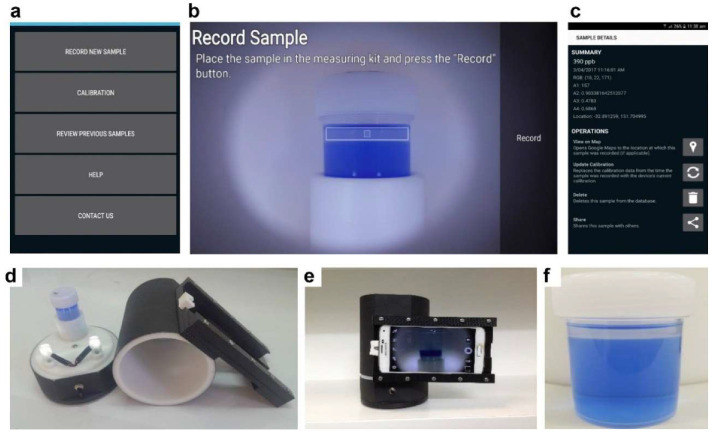
Snapshots (**a**–**c**) of the app, photos of the reading kit (**d**,**e**), and sample (**b**,**f**). In (**b**), the reading screen’s rectangle zone is positioned over the non-aqueous phase layer and the central part (inner square) is the color reading area. In (**d**), two white LEDs are on and a container of 25 mL containing the sample is positioned in a sample holder. In (**e**), the smartphone holder is shown holding a smartphone in the reading position. Note the different orientations (portrait or landscape) of the smartphone in (**a**–**c**,**e**). (For interpretation of the references to color in this figure legend, the reader is referred to the web version of this article). Reproduced with permission from Fang et al. [75].

As touched on in the optical sensor discussion, the concentration change of target analytes can be easily transformed into color changes, which can often be observed by the naked eye alone, thereby negating the need for sophisticated instruments. An observed trend in sensor technology is the use of these available optical properties in a smartphone-based sensor. This technology has made a significant breakthrough in the field of on-site trace analyses of constituents, such as halogenic compounds, heavy metals, hydrogen ions, biomaterials, organic and inorganic substances, and recently, PFASs [74]. Fang et al. demonstrated the use of an app-based monitoring tool for the detection of PFOA concentrations in spiked tap/groundwater [75]. Based on the concept that anionic surfactants, such as PFOA and PFOS, can interact with cationic dyes to form ion pairs, they utilized liquid-phase extraction (LPE) to extract the hydrophobic ion pair of a dye to an organic phase [75]. The proposed app-based sensor relies on the camera of a smartphone to “read” the color (RGB) of the organic phase (Figure 4). The intensity of the color is carefully corrected, linked to the concentration of the ion pair, and then applied to determine the concentration of PFOA. They achieved concentration outputs with a standard deviation of <10% in the 10–1000 ppb range and a LOD as low as 0.5 ppb [75]. However, this method requires both LPE and SPE to first be performed to pre-concentrate the sample and mitigate potential background interference, which can increase the measurement time to beyond 3 h. In addition, the LOD achieved, although low, is still higher than the USEPA recommended level of 0.07 ppb [10].

The work completed by Fang et al. demonstrates the opportunity to use smartphones as a unique platform to increase sensor availability [75]. Diving further into the field of smart technology, “smart cities” promote the concept of employing a diverse range of sensors to accumulate detailed information about changes in the environment. A study completed by Cennamo et al. contributed to this discussion by developing a low-cost, portable sensor system with the intention of connecting it to the Internet of things (IoT) in future works [104]. The proposed sensor utilizes POFs and MIPs on a surface plasmon resonance (SPR) platform for the detection of perfluorobutanesulfonic acid (PFBS), which is a pollutant that is difficult to adsorb with common adsorbent media. Their results showed that the MIP promoted adsorption and that the SPR-POF technique could detect relative refraction index variations, achieving an inverse correlation between the concentration of PFBS in the sample and the refractive index value of the MIP layer. Another advantage of MIPs that is particularly significant to overarching water monitoring in smart cities was also made clear, namely, the concept that several MIP receptors can be deposited on POF platforms to detect various target analytes, which is transformative.

Further, Cennamo et al. suggested that the proposed optical fiber configuration can be directly connected to an online platform via interface software for Raspberry Pi, which has the potential to analyze and display the sensor’s data [104]. They used a single-board computer with software (developed by free SeaBreeze opensource driver for Advanced reduced instruction set computer (RISC) machine (ARM) microcontroller connectivity), which includes several dedicated functions to guide the automated processing of the data gathered from the sensor [104]. A possible future work will expand on the requirements necessary to connect the sensor’s data to the internet, such as data transmission, storage, management, and final presentation, as well as the effects of merging PFAS-detecting sensors with smart technology in environmental quality monitoring. Although there are not many additional discussions on PFASs determination using smart technology at this point, it is understood that this is a promising direction within this field. Like the general emergence of sensor determination in environmental matrices, its pairing with smart technology has also surfaced with significant popularity.

## 5. Concluding Remarks

As regulations on PFASs have recently evolved, the urgency regarding their determination methods has increased for protecting public health. Given that PFASs, as forever chemicals, are everywhere, more data from water, soils, and agriculture are needed to better understand the exposure pathways and health outcomes, and to develop associated risk mitigation actions [4]. Our work reviewed the current methods of determination and presented the paradigm shift in the literature from laboratory-based analyses toward in situ sensor technologies. This promotes remote analyses at low costs, which provides many benefits for continuous environmental water quality monitoring and management. The direction of sensor technology supports the development of a network of sensors that is capable of working in swarms. PFAS-detecting sensors may be utilized in a network of sensors to enhance the spatiotemporal data gathered regarding water bodies of interest, thus enhancing decision-making. Sixty years of general remote sensing research has prompted use in water management systems, yet certain limitations have hampered their full-scale adoption [116].

Previously, environmental sensors for water quality monitoring tended to be unintelligent, connected directly into control systems, and static. However, the introduction of smart technology allows for wireless sensor networks that can deploy and locate themselves; efficiently collect, process, and transmit data; and potentially provide a control response to the environment. The focal points of PFASs detection using smart technology include determining which properties will be utilized for detection, automatic ranging, remote calibration, advances in microprocessors, and new algorithms [117]. The integration of wireless sensor networks with cloud computing then provides a method of sensor virtualization, which is helpful for transmitting, storing, and sharing data [118].

## Figures and Tables

**Table 1:** Current analytical tools for per- and poly-fluoroalkyl substance (PFAS) measurements in various environmental matrices.

PFAS	Environmental/Laboratory Media	Concentration Spiked/Measured/Detected	Sample Extraction/Analytical Tool	Reported Detection Limits	Ref.
PFHxA, PFHpA, PFOA, PFNA, PFDA, PFUnDA, PFDoDA, PFHxS, PFOS, PFDS	Rain, snowmelt, and stream water	Up to 1691 pg/L	SPE ^a^-UPLC ^b^-MS ^c^	MDL ^d^: 3–76 pg/L	[21]
PFBA, PFPeA, PFHxA, PFHpA, PFOA, PFNA, PFDA, PFUnDA, PFDoDA, PFBS, PFHxS, PFOS, PFOSA	Biosolids	Up to 403 ± 127 ng/g dry weight	SPE-LC-MS/MS ^e^	MDL: 0.03 and 0.14 ng/g	[22]
PFHxA, PFHpA, PFOA, PFNA, PFDA, PFUnA, PFDoA, PFOS, PFDS, PFOSA, *N*-EtPFOSA,	WWTP effluent	Up to 21 ng/L	SPE/LLE ^f^ LC-MS/MS	MDL: 0.25–0.64 ng/L	[23]
PFHxA, PFHpA, PFOA, PFNA, PFDA, PFUnA, PFDoA, PFOS	River water, seawater, and WWTP influent and effluent	Up to 401 ng/L	IPE ^g^-LC-MS/MS	LOD ^h^: 0.2–20 ng/L	[24]
PFBA, PFPA, PFHA, PFOA, PFNA, PFDA	Ultrapure water, tap water, river water, and wastewater	0.0025–150 μg/L	MMF ^i^-SPME ^j^–HPLC ^k^–MS/MS	LOD: 0.40–4.40 ng/L	[25]
PFHxA, PFOA, PFNA, PFDA, PFUnDA, PFDoDA, PFTrDA, PFTeDA, PFHxDA, PFODA, PFBS, PFPeS, PFHxS, PFHpS, PFOS, PFNS, PFDS, C8 Cl-PFESA, C10 Cl-PFESA, C12 Cl-PFESA	Tap water, river water, and urine samples	Up to 120 ng/L	DLLME ^l^-LC-MS/MS	MDL: 0.6–8.7 ng/L	[26]
PFOA, PFOS	Honey, Milli-Q	0.103–0.223 ng/g	*d*-SPE ^m^-UHPLC ^n^-MS/MS	LOD: 0.016–0.040 µg/kg LOQ ^o^: 0.052–0.134 µg/kg	[27]
PFOA, PFOS, PFHpA, PFNA, PFDA, PFHxS	Drinking water, tap water, pond water, and seawater	Up to 1000 ng/L	SPE-LC-MS/MS	LOD: 0.01–1.15 ng/L LOQ: 0.03–3.85 ng/L	[28]
PFBA, PFPeA, PFHxA, PFHpA, PFOA, PFNA, PFDA, PFUnA, PFDoA, PFTeDA, PFBS, PFHxS, PFOS	Surface water	Up to 898 ng/L	*µ*SPE ^p^-LC-MS/MS	LOD: 0.29–6.6 ng/L	[29]
PFOA PFOS	Tap water, river water	Up to approximately 35 ng/L	SPME–LC–MS	LOD: 1.5 ng/L for PFOA and 3.2 ng/L for PFOS	[30]
PFHxI, PFOI, 4:2 FTI, 6:2 FTOH, 6:2 FTI, 8:2 FTOH, 6:2 FTAC, 8:2 FTI, 10:2 FTOH, 8:2 FTAC, 8:2 FTMAC, MeFOSA, EtFOSA	Tap water, surface water, and sediments	102–246 ng/L in water, 1.1–5.7 ng/g in sediment	HS ^q^-SPME-GC ^r^/MS	LOQ: 20–100 ng/L for water, 1–3 ng/g for sediments	[31]
PFOS	Tap water, river water, and well water	10–500 ng/L	VALLME ^s^-LC-MS	LOD: 1.6 ng/L	[32]
PFHpA, PFOA, PFNA, PFDA, PFUdA, PFDoA, PFTrDA, PFTeDA	Canal water	< LOQ	BHF-LPME ^t^-LC-MS/MS	LOD: 0.40–57.4 ng/L LOQ: 1.25–224 ng/L	[33]
PFOA	Deionized (DI) water	50–100 mg/L	HPLC	LOD: 1 mg/L	[34]
PFOS	Water:methanol (1:4)	1–500 mg/L	UPLC-MS	LOD: 0.4 ng/mL LOQ: 1 ng/mL	[35]
PFBA, PFPeA, PFHxA, PFHpA, PFOA, PFNA, PFDA, PFUnDA, PFDoDA, PFTrDA, PFTeDA, PFHxDA, PFOcDA, PFBS, PFHxS, PFOS, PFDS, FOSA, FOSAA	River water, coastal wastewater, and wastewater treatment plant (WWTP) effluent	13.1–69,238 ng/L	SPE-HPLC-MS/MS	LOQ: 0.05–0.22 ng/L	[36]
PFOA, selected short-chain PFCAs	Ultrapure/DI water	100 mg/L	HPLC-MS/MS	LOD: 0.01–12.3 µg/L LOQ: 0.20–53.6 µg/L	[37]
PFOA, PFOS, PFBA, PFPeA, PFHxA, PFNA, PFDA, PFBS, PFHxS	DI water and artificial ground water	1 µg/L	LC-MS/MS	LOQ: 10–20 ng/L	[38]
PFBA, PFPeA, PFHxA, PFHpA, PFOA, PFNA, PFDA, PFUnA, PFBS, PFHxS, PFOS	Artificial groundwater	0.5–1000 μg/L	LC-MS/MS	LOQ: 0.02–0.5 ng/g (soil), 2–9 ng/L (aqueous samples)	[39]
PFOA	Ultrapure water, tap water, and river water	0.12–2.42 μmol/L	LC-MS/MS	N/R ^u^	[40]
PFOS, PFOA	Riverwater and samples from a drinking water treatment plant (from different units and processes)	<1.1–11,120 ng/L	LLE-LC-MS/MS	LOD: 0.4–1.9 ng/L LOQ: 1.1–4.2 ng/L	[41]
PFHxA, PFHpA, PFOA, PFNA, PFDA, PFHxS, PFOS	Biosolids and biosolid-amended soils	Up to 483 ng/g	LC-MS/MS	LOQ: 0.02–0.5 ng/g	[42]
PFOA, PFOS, PFHxA, PFHpA, PFNA, PFDA, PFBS, PFHxS	WWTP effluent and reclaimed water via advanced treatment (membrane, advanced oxidation)	Up to 39 ng/L	HPLC-MS/MS	0.4–1.5 ng/L (reporting limit)	[43]
PFOS, PFOA, PFDA, PFUnA, PFDoA, PFTA, PFDS, PFNA, PFHxS, *N*-EtFOSAA, *N*-MeFOSAA, FOSAA	Sediments and domestic sludge	Up to 3370 ng/g	SPE-HPLC-MS/MS	LOD: 0.041–2.2 ng/g	[44]
PFOS	Semiconductor wastewater	0.5–1500 ppm	LC–MS/MS	N/R	[45]
PFOS, PFOA	Different units/processes of a sewage treatment plant, sludge	7.9–113.9 ng/L	SPE-HPLC-MS/MS	LOD: 0.1–0.5 ng/L in wastewater, 1–5 ng/g in sludge LOQ: 0.25–1 ng/L in wastewater, 2–8 ng/g in sludge	[46]
PFOS, PFOA, PFBuS, PFHxS, PFHpS, PFDS, PFBA, PFPeA, PFHxA, PFHpA, PFNA, PFDA, PFUnDA, PFDoA, PFTrA, PFTA, FOSA, N-MeFOSA, N-EtFOSA	Wastewater, sludge, and sediment	1–49.9 ng/L	UPLC-MS/MS	MDL: 0.57–2.86 ng/L for water, 0.14–1.43 ng/g for sludge, 0.03–0.14 ng/g for sediment	[47]
PFOA	Lake water, river water, groundwater, and tap water	0.1–92 ng/L	SPE-LC-MS/MS	LOQ: 0.1–1.0 ng/L	[48]
PFOS, PFOA	DI water	0.04–0.4 mM	HPLC	LOD: 0.11–0.18 mg/L	[49]
PFBA, PFPeA, PFBS, PFHxA, PFHpA, PFHxS, PFOA, PFHpS, PFNA, PFOS, PFDA	Different stages in drinking water and wastewater treatment plants	Up to 195 ± 12.8 ng/L	SPE-HPLC-MS/MS	LOD: 0.02–0.10 ng/L for water, 0.02–0.04 ng/g for sludge LOQ: 0.06–0.33 ng/L for water, 0.066–0.14 ng/g for sludge	[50]

^a^ SPE: Solid-phase extraction; ^b^ UPLC: Ultra-performance liquid chromatography; ^c^ MS: Mass spectrometry; ^d^ MDL: Method detection limit; ^e^ LC-MS/MS: Liquid chromatography–tandem mass spectrometry; ^f^ LLE: Liquid–liquid extraction; ^g^ IPE: Ion-pair extraction; ^h^ LOD: Limit of detection; ^i^ MMF: multiple monolithic fiber; ^j^ SPME: Solid-phase microextraction; ^k^ HPLC: High-performance liquid chromatography; ^l^ DLLME: Dispersive liquid–liquid microextraction; ^m^ *d*-SPE: Dispersive solid phase extraction; ^n^ UHPLC: Ultrahigh performance liquid chromatography; ^o^ LOQ: Limit of quantification; ^p^ *µ*SPE: micro-SPE; ^q^ HS: Headspace; ^r^ GC: Gas chromatography; ^s^ VALLME: Vortex-assisted liquid–liquid microextraction; ^t^ BHF–LPME: Bundled hollow fiber array-liquid-phase microextraction; ^u^ N/R: Not reported.

**Table 2:** Methods of sensor detection of various PFAS species and their respective detection limits.

Detection Method	Species Detected	LOD	Ref.
MIP-coated Au electrode	PFOS	0.04 nM	[78]
MIP-coated TiO_2_ nanotubes	PFOS	86,000 ng·L^−1^	[81]
MIP-coated plastic optical fiber platform	PFOA	130 ng·L^−1^	[82]
MIP fluorescence sensor	PFOS	5.57 µg·L^−1^	[79]
Electrochemical biosensor using an enzymatic biofuel cell (BFC)	PFOS	1.6 nM	[83]
Bubble-nucleation-based electrochemical sensor	PFOA, PFOS	20 nM (or 30 µg·L^−1^)	[80]
Surface plasmon resonance (SPR) optical fiber biosensor	PFOA, PFOS	0.21 µg·L^−1^	[84]
Potentiometric detection using MIP-coated pencil lead	PFOA, PFOS, 6:2FTS	10 µM–10 mM	[76]
Potentiometric detection using ion-selective electrodes (ISEs)	PFO^− a^, PFOS	0.07–1.0 µg·L^−1^	[77]
Potentiometric detection using metal-organic framework and interdigitated electrodes	PFOS	0.5 ng·L^−1^	[85]

^a^ PFO^−^: perfluorooctanoate.

**Table 3:** Summary of the various optical and fluorescence sensors for PFAS detection.

Matrix	Detector	Working Range	LOD	Note	Ref.
D-shaped POF ^a^	Optical density	0–200 ppb	0.21 ppb	D-shaped POF was characterized using a very simple and low-cost experimental setup based on an LED and two photodetectors.	[105]
Polymer-AuNP ^b^	Naked eye	-	100 ppm	PFOA detached polystyrene from AuNP surface	[106]
SAM-AuNP	Naked eye	10–1000 ppb	10 ppb	The colorimetric assay for the detection of PFCs, but the long chain of PFCs (>7) is discerned.	[107]
QD ^c^-bioassay	Fluorescence	2.7–7.5 ppt	2.5 ppt	Bioassay based on PFOS binding to PPARα	[108]
Bio-AuNP	Optical density	50 ppt–500 ppb	5 ppt	Bioassay based on the silver enhancement of AuNP and interaction among ligands, PPARα, and PPRE	[109]
MIP-C_3_N_4_	Electrochemiluminescence	0.02–400 ppb	0.01 ppb	PFOA is efficiently oxidized by the electro-generated (SO_4_^·^^−^); thus, this sensor is highly sensitive to PFOA	[110]
MPA ^d^-QD	Fluorescence	200–16,000 ppb	120 ppb	PFOA strongly quenched the fluorescence emission of the MPA-CdS QDs	[111]
App-based	Smartphone camera	10–1000 ppb	0.5 ppb	PFOA sensing. Requires SPE ^e^ pretreatment of samples	[75]
SPR-POF-MIP	Optical density	-	<1 ppb	PFBS sensing	[104]

^a^ POF: Plastic optical fiber, ^b^ AuNP: Gold nanoparticle, ^c^ QD: Quantum dot, ^d^ MPA: 3-mercaptopropionic acid, ^e^ Solid-phase extraction.

## Abbreviations

Cl-PFESA	Chlorinated polyfluoroether sulfonic acid
EtFOSA	*N*-Ethyl perfluorooctane sulfonamide
FOSAA	Perfluorooctane sulfonamidoacetate
FTAC	Fluorotelomer acrylate
FTI	Fluorotelomer iodide
FTMAC	Fluorotelomer methacrylate
FTOH	Fluorotelomer alcohol
MeFOSA	*N*-Methyl perfluorooctane sulfonamide
*N*-EtFOSA/*N*-EtPFOSA	*N*-ethylperfluorooctane sulfonamide
*N*-EtFOSAA	*N*-ethylperfluorooctanesulfonamido acetic acid
*N*-MeFOSAA	*N*-methylperfluorooctanesulfonamido acetic acid
PFAAs	Perfluoroalkyl acids
PFBA	Perfluorobutyric acid
PFBS	Perfluorobutane sulfonic acid
PFBuS	Perfluorobutane sulfonic acid (PFBuS)
PFCA	Perfluoroalkyl carboxylic acids
PFDA	Perfluorodecanoic acid
PFDoA/PFDoDA	Perfluorododecanoic acid
PFDS	Perfluorodecane sulfonate
PFDS	Perfluorodecane sulfonate
PFHA	Perfluoroheptanoic acid
PFHpA	Perfluoroheptanoic acid
PFHpS	Perfluoropentane sulfonate
PFHxA	Perfluorohexanoic acid
PFHxDA	Perfluorohexadecanoic acid
PFHxI	Perfluorohexyl iodide
PFHxS	Perfluorohexanesulfonate
PFNA	Perfluorononanoic acid
PFNA	Perfluorononanoic acid
PFNS	Perfluorononane sulfonate
PFO	perfluorooctanoate
PFOA	Perfluorooctanoic acid
PFOcDA	Perfluorooctadecanoic acid
PFODA	Perfluorooctadecanoic acid
PFOI	Perfluorooctyl iodide
PFOS	Perfluorooctanesulfonic acid
PFOSA/FOSA	Perfluorooctanesulfonamide
PFPA	Perfluoropentanoic acid
PFPeA	Perfluoropentanoic acid
PFPeS	Perfluoropentane sulfonate
PFTA	perfluorotetradecanoic acid
PFTeDA	Perfluorotetradecanoic acid
PFTrDA	Perfluorotridecanoic acid
PFUnA/PFUnDA/PFUdA	Perfluoroundecanoic acid
